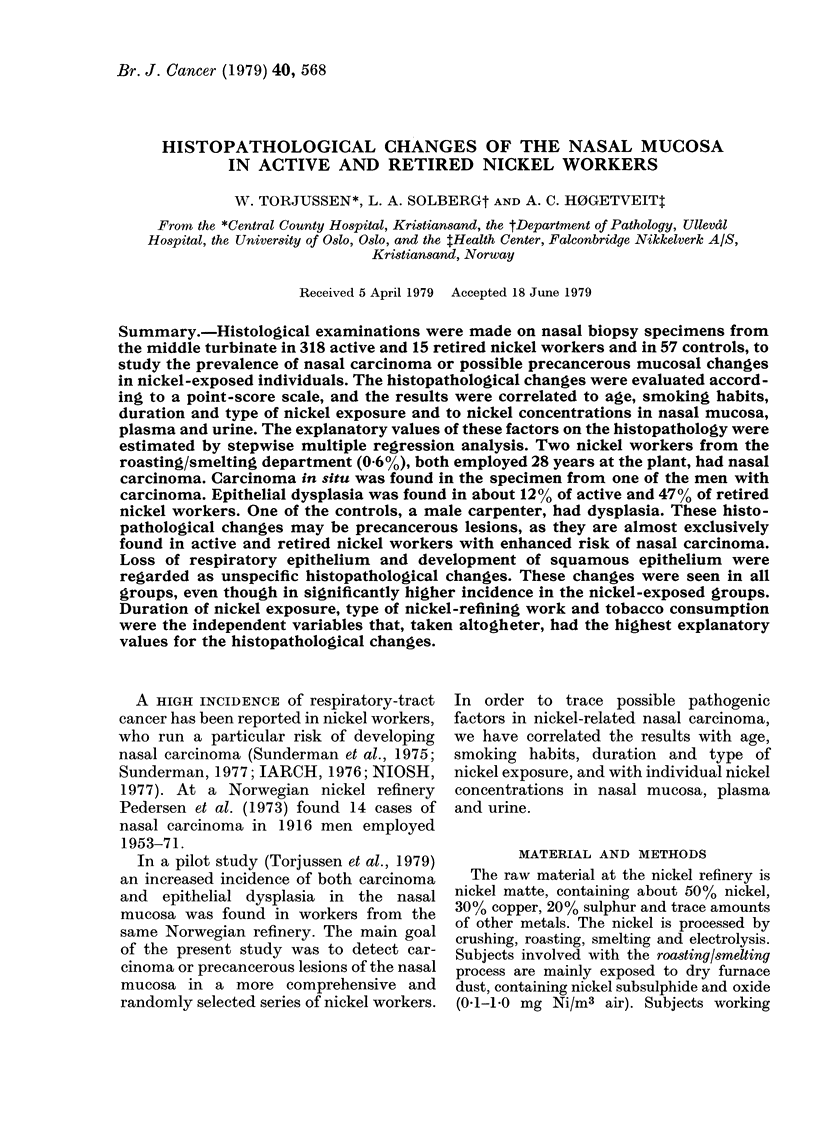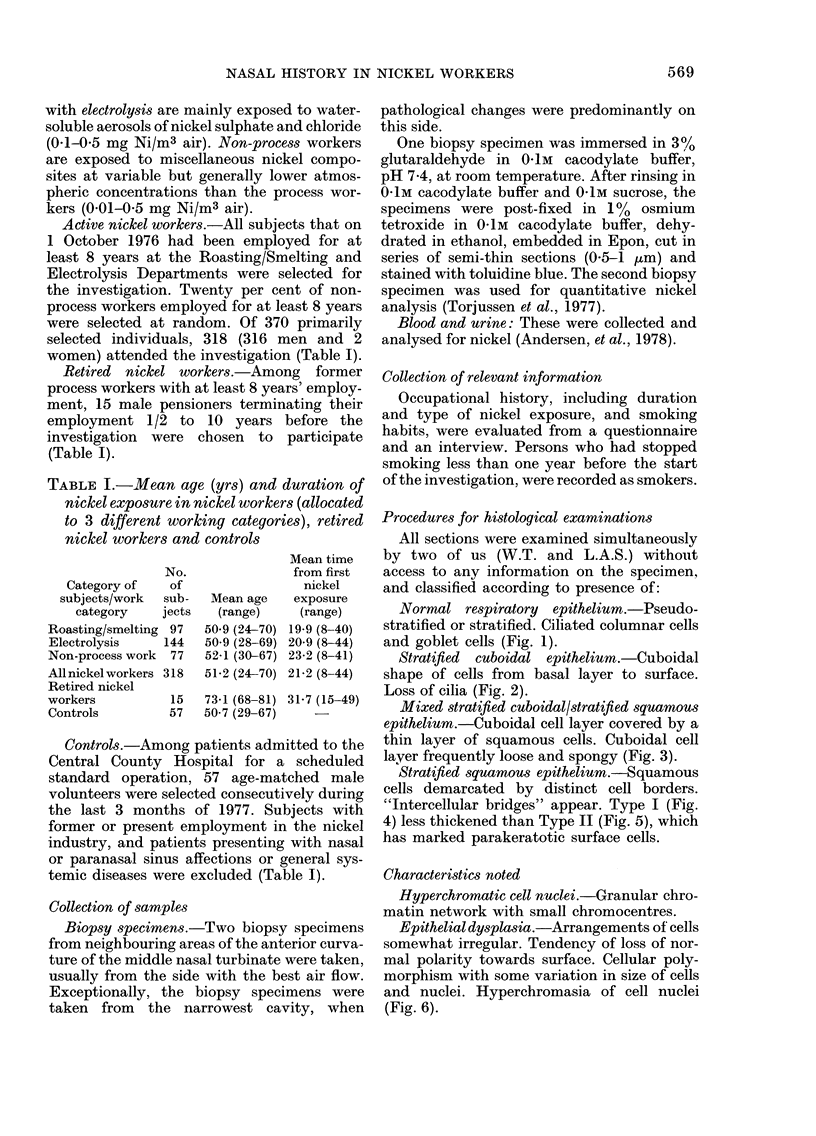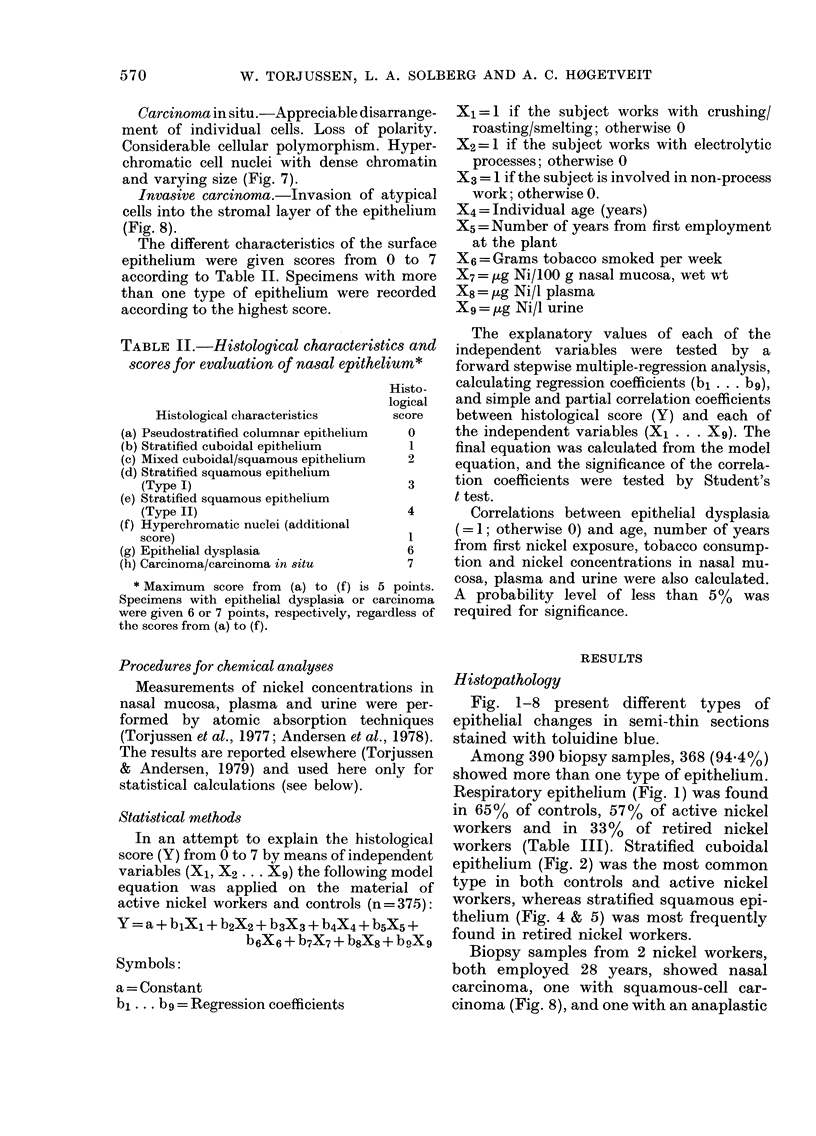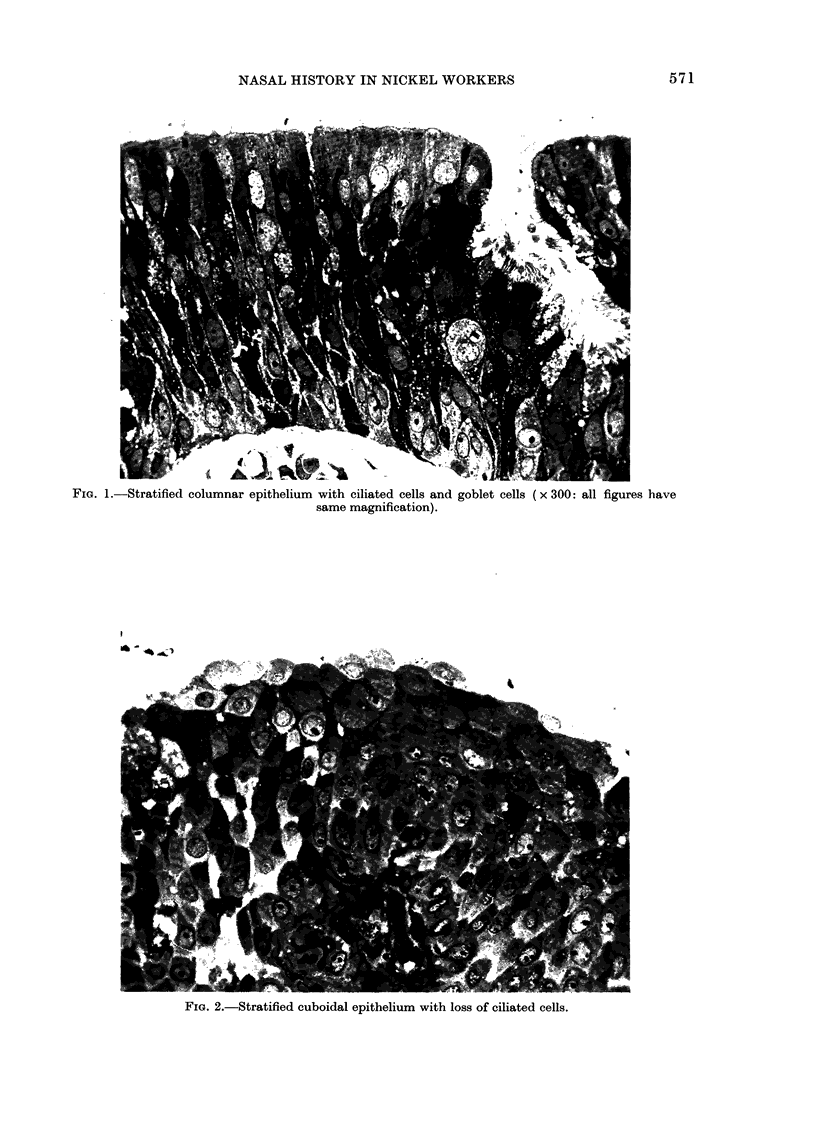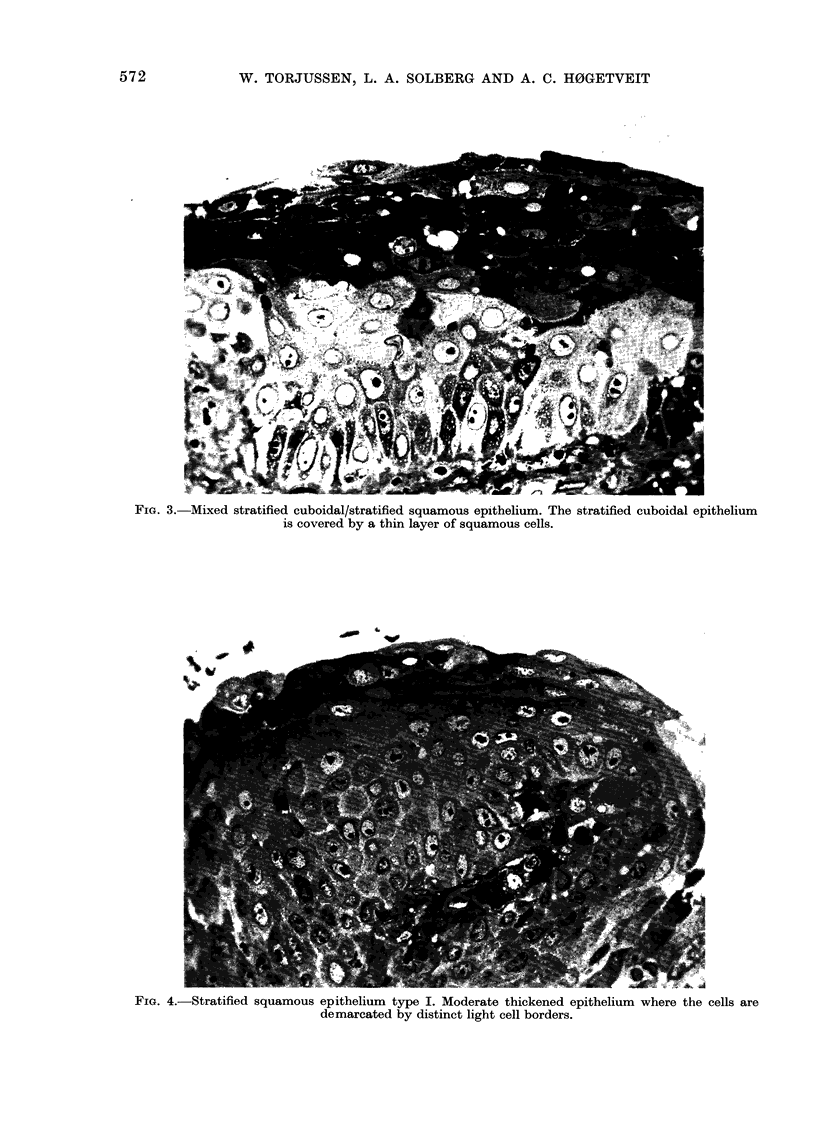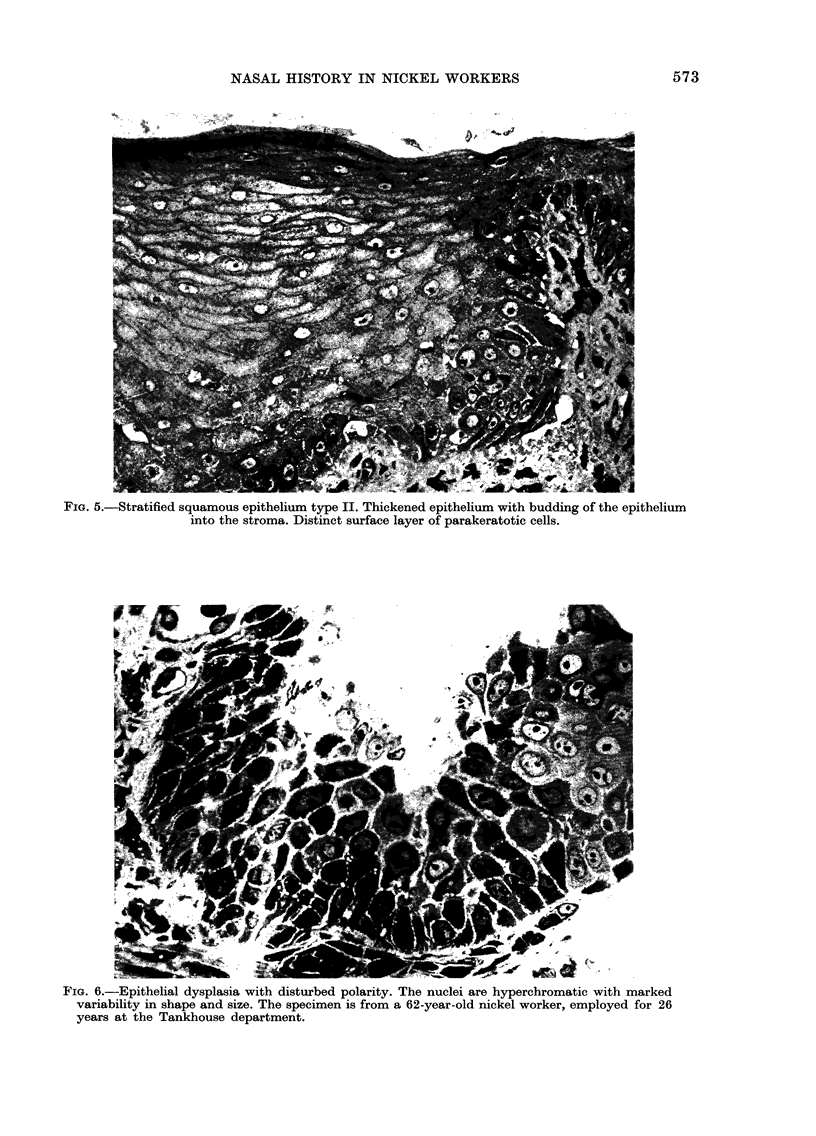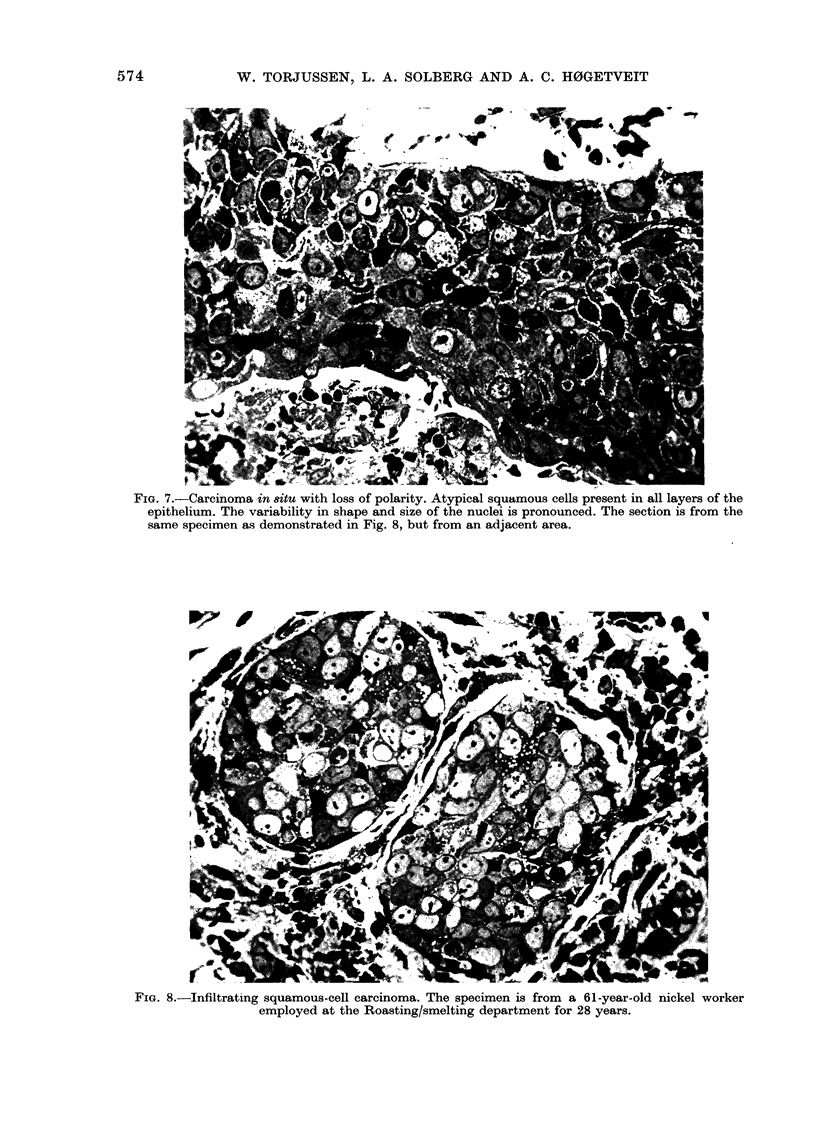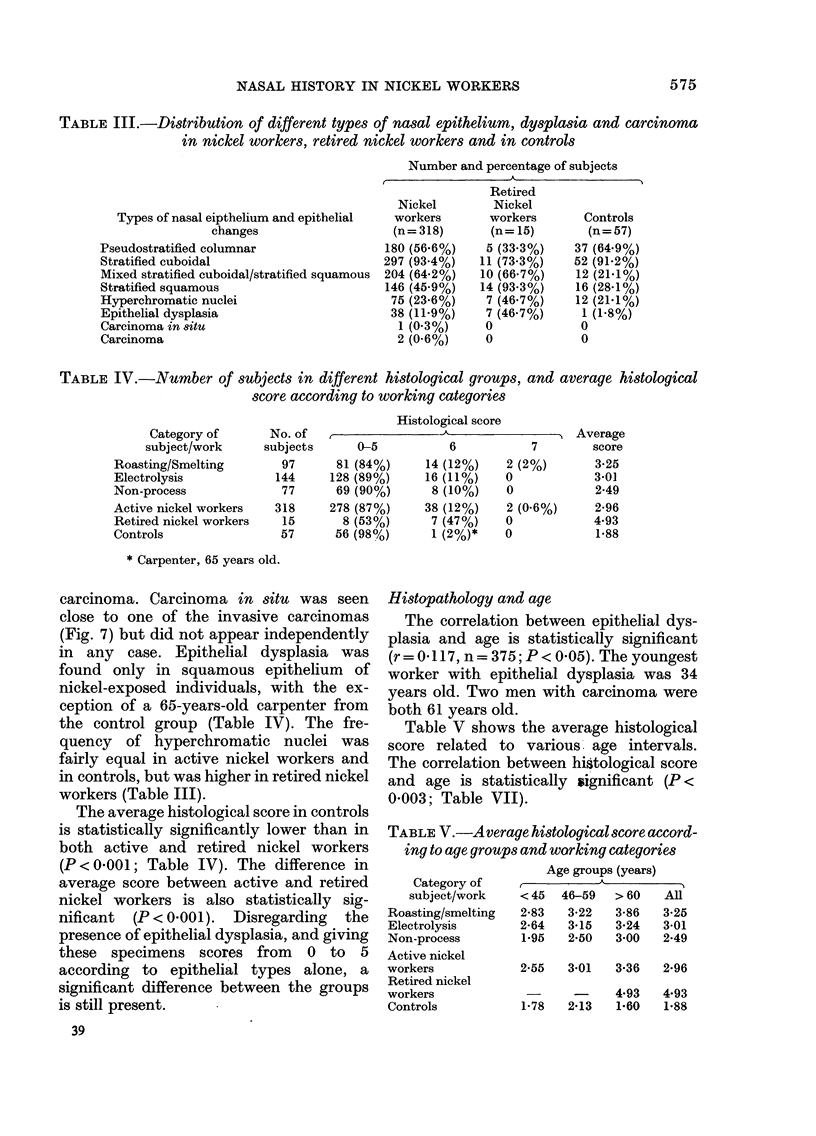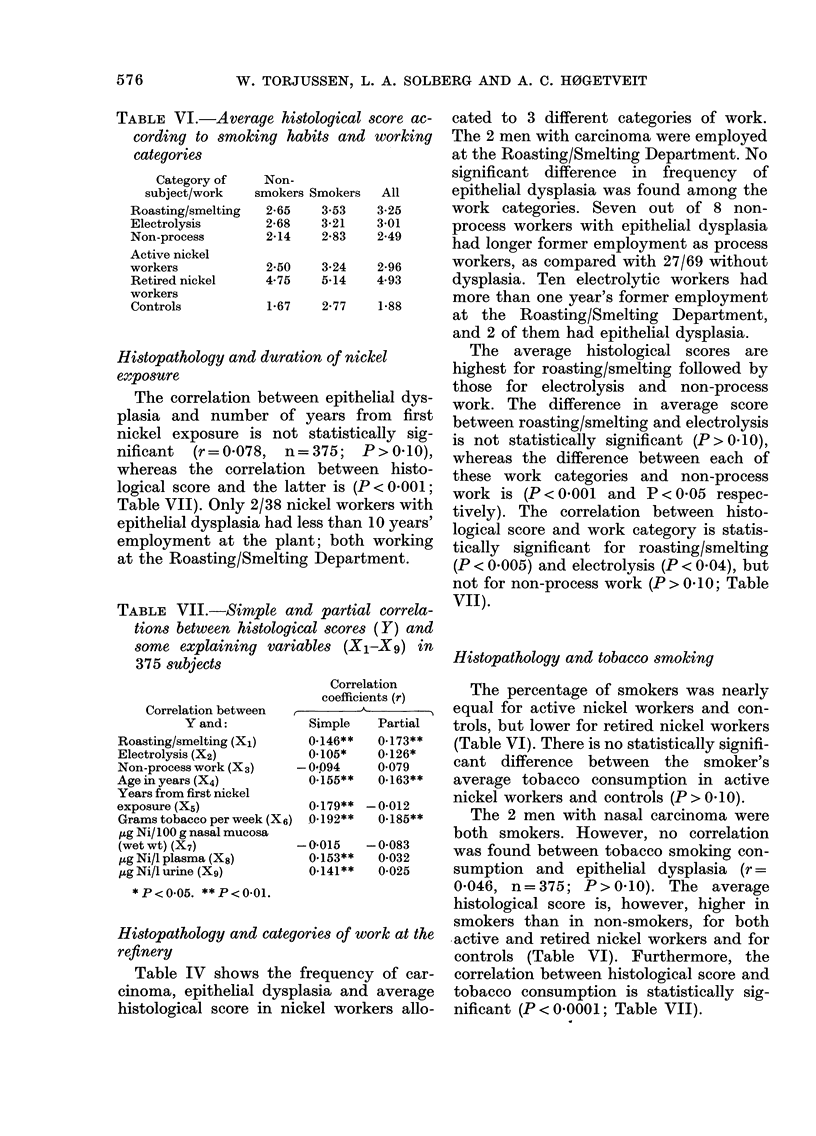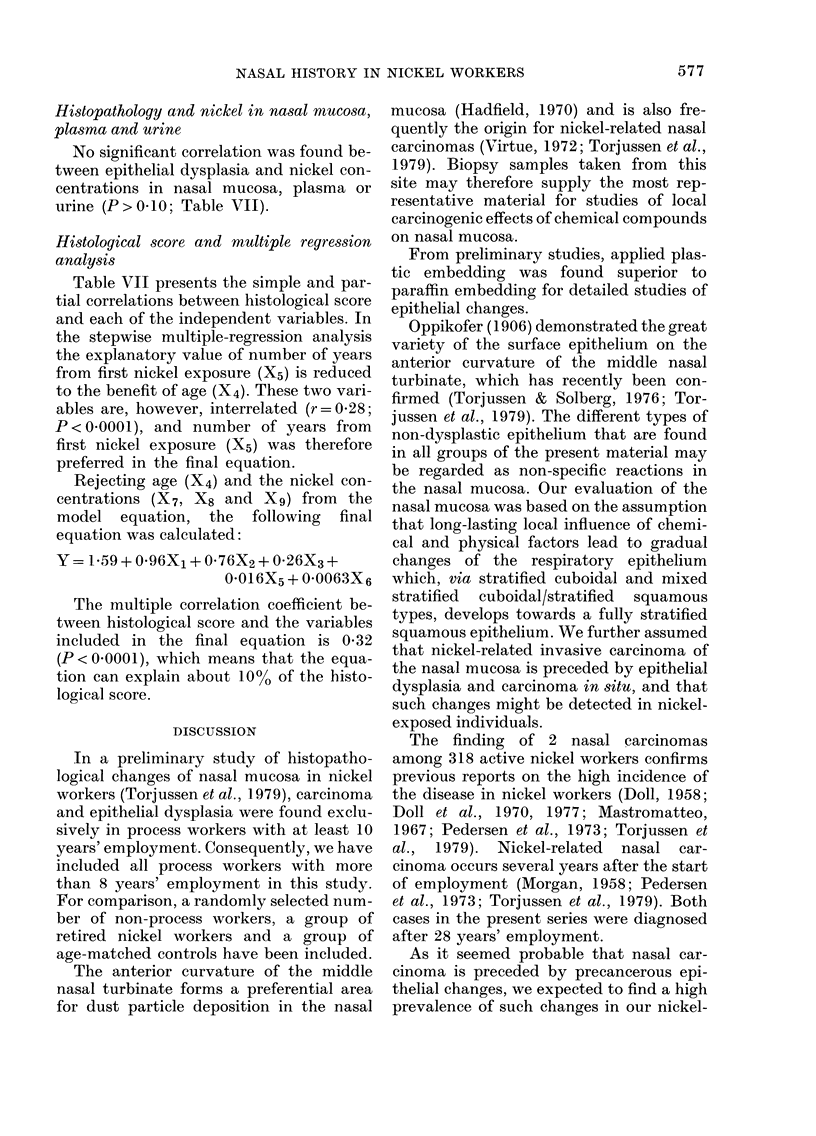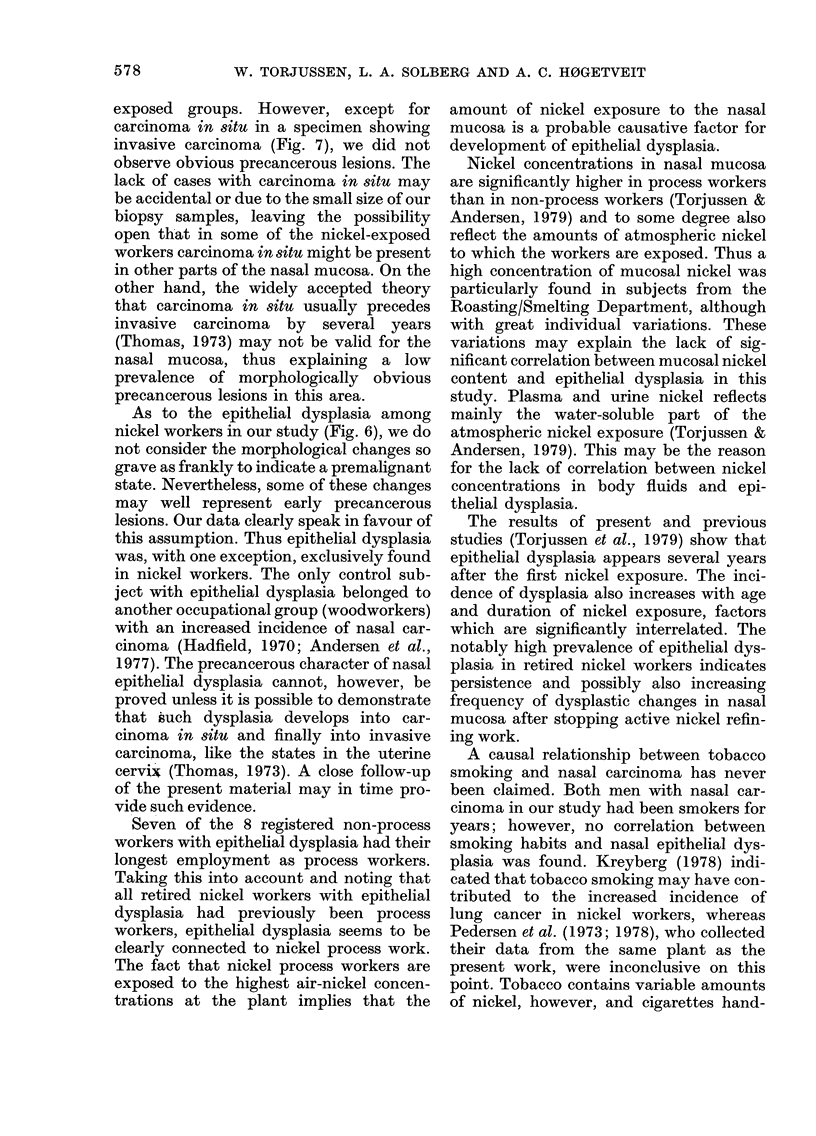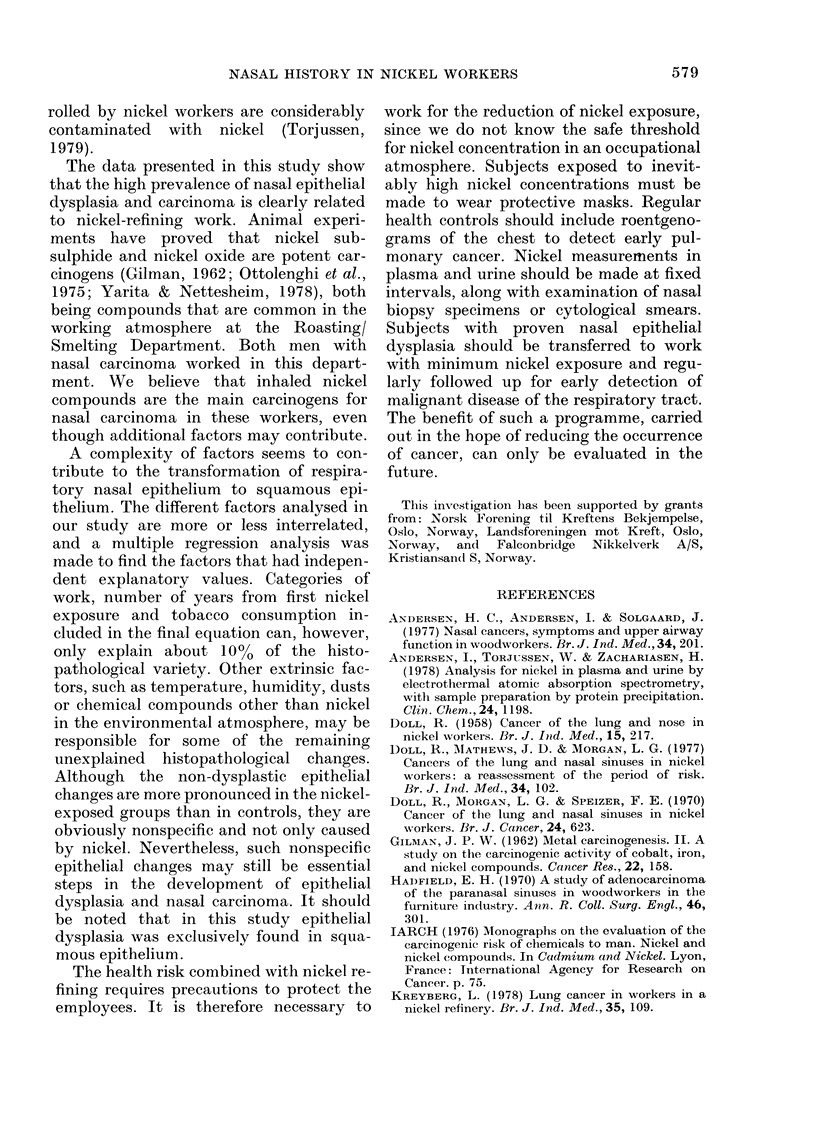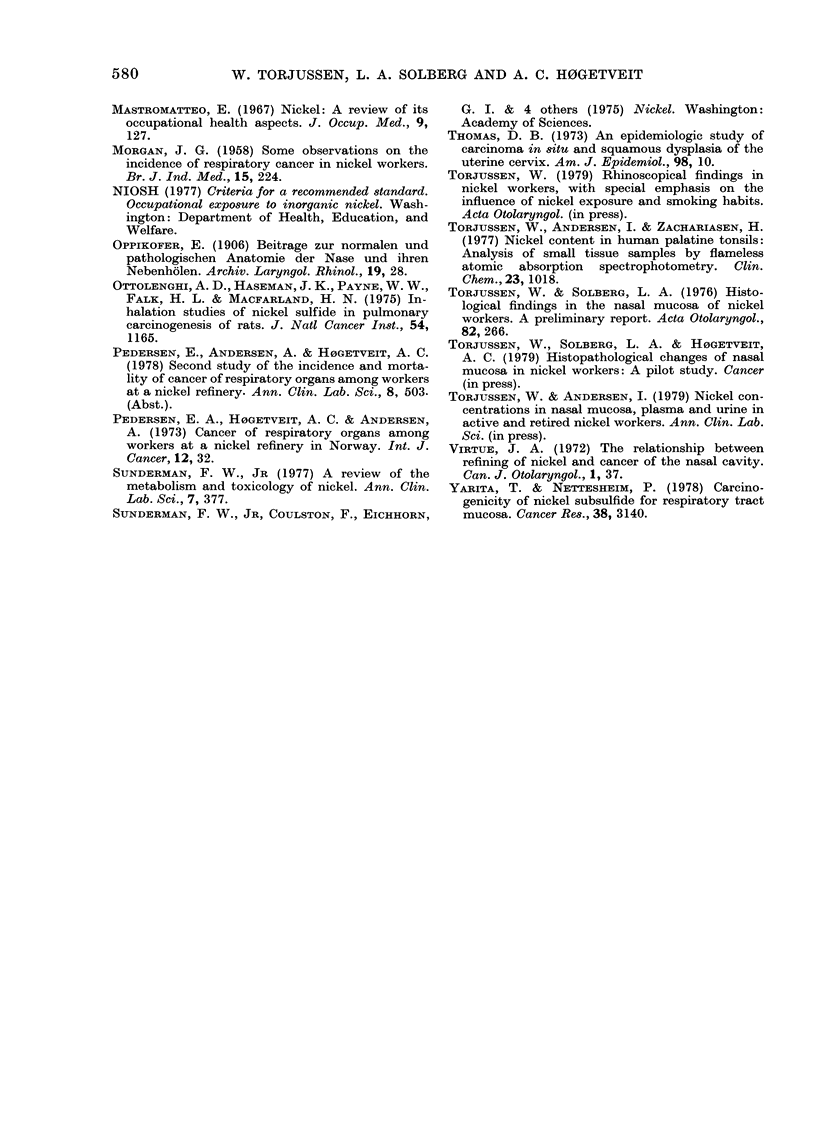# Histopathological changes of the nasal mucosa in active and retired nickel workers.

**DOI:** 10.1038/bjc.1979.222

**Published:** 1979-10

**Authors:** W. Torjussen, L. A. Solberg, A. C. Høgetveit

## Abstract

**Images:**


*Br. J. Cancer* (1979) **40**, 568

### HISTOPATHOLOGICAL CHANGES OF THE NASAL MUCOSA IN ACTIVE AND RETIRED NICKEL WORKERS

W. TORJUSSEN*, L. A. SOLBERG† AND A. C. HØGETVEIT‡

*From the *Central County Hospital, Kristiansand, the †Department of Pathology, Ullevål Hospital, the University of Oslo, Oslo, and the ‡Health Center, Falconbridge Nikkelverk A/S, Kristiansand, Norway*

Received 5 April 1979 Accepted 18 June 1979

**Summary.—Histological examinations were made on nasal biopsy specimens from the middle turbinate in 318 active and 15 retired nickel workers and in 57 controls, to study the prevalence of nasal carcinoma or possible precancerous mucosal changes in nickel-exposed individuals. The histopathological changes were evaluated according to a point-score scale, and the results were correlated to age, smoking habits, duration and type of nickel exposure and to nickel concentrations in nasal mucosa, plasma and urine. The explanatory values of these factors on the histopathology were estimated by stepwise multiple regression analysis. Two nickel workers from the roasting/smelting department (0·6%), both employed 28 years at the plant, had nasal carcinoma. Carcinoma *in situ* was found in the specimen from one of the men with carcinoma. Epithelial dysplasia was found in about 12% of active and 47% of retired nickel workers. One of the controls, a male carpenter, had dysplasia. These histopathological changes may be precancerous lesions, as they are almost exclusively found in active and retired nickel workers with enhanced risk of nasal carcinoma. Loss of respiratory epithelium and development of squamous epithelium were regarded as unspecific histopathological changes. These changes were seen in all groups, even though in significantly higher incidence in the nickel-exposed groups. Duration of nickel exposure, type of nickel-refining work and tobacco consumption were the independent variables that, taken altogheter, had the highest explanatory values for the histopathological changes.**

A HIGH INCIDENCE of respiratory-tract cancer has been reported in nickel workers, who run a particular risk of developing nasal carcinoma (Sunderman *et al.*, 1975; Sunderman, 1977; IARCH, 1976; NIOSH, 1977). At a Norwegian nickel refinery Pedersen *et al.* (1973) found 14 cases of nasal carcinoma in 1916 men employed 1953–71.

In a pilot study (Torjussen *et al.*, 1979) an increased incidence of both carcinoma and epithelial dysplasia in the nasal mucosa was found in workers from the same Norwegian refinery. The main goal of the present study was to detect carcinoma or precancerous lesions of the nasal mucosa in a more comprehensive and randomly selected series of nickel workers. In order to trace possible pathogenic factors in nickel-related nasal carcinoma, we have correlated the results with age, smoking habits, duration and type of nickel exposure, and with individual nickel concentrations in nasal mucosa, plasma and urine.

### MATERIAL AND METHODS

The raw material at the nickel refinery is nickel matte, containing about 50% nickel, 30% copper, 20% sulphur and trace amounts of other metals. The nickel is processed by crushing, roasting, smelting and electrolysis. Subjects involved with the *roasting/smelting* process are mainly exposed to dry furnace dust, containing nickel subsulphide and oxide (0·1–1·0 mg $Ni/m^3$ air). Subjects working with *electrolysis* are mainly exposed to water-soluble aerosols of nickel sulphate and chloride (0·1–0·5 mg Ni/m$^3$ air). *Non-process* workers are exposed to miscellaneous nickel composites at variable but generally lower atmospheric concentrations than the process workers (0·01–0·5 mg Ni/m$^3$ air).

*Active nickel workers.*—All subjects that on 1 October 1976 had been employed for at least 8 years at the Roasting/Smelting and Electrolysis Departments were selected for the investigation. Twenty per cent of non-process workers employed for at least 8 years were selected at random. Of 370 primarily selected individuals, 318 (316 men and 2 women) attended the investigation (Table I).

*Retired nickel workers.*—Among former process workers with at least 8 years' employment, 15 male pensioners terminating their employment 1/2 to 10 years before the investigation were chosen to participate (Table I).

TABLE I.—*Mean age (yrs) and duration of nickel exposure in nickel workers (allocated to 3 different working categories), retired nickel workers and controls*

| Category of subjects/work category | No. of subjects | Mean age (range) | Mean time from first nickel exposure (range) |
|---|---|---|---|
| Roasting/smelting | 97 | 50·9 (24–70) | 19·9 (8–40) |
| Electrolysis | 144 | 50·9 (28–69) | 20·9 (8–44) |
| Non-process work | 77 | 52·1 (30–67) | 23·2 (8–41) |
| All nickel workers | 318 | 51·2 (24–70) | 21·2 (8–44) |
| Retired nickel workers | 15 | 73·1 (68–81) | 31·7 (15–49) |
| Controls | 57 | 50·7 (29–67) | — |

*Controls.*—Among patients admitted to the Central County Hospital for a scheduled standard operation, 57 age-matched male volunteers were selected consecutively during the last 3 months of 1977. Subjects with former or present employment in the nickel industry, and patients presenting with nasal or paranasal sinus affections or general systemic diseases were excluded (Table I).

### *Collection of samples*

*Biopsy specimens.*—Two biopsy specimens from neighbouring areas of the anterior curvature of the middle nasal turbinate were taken, usually from the side with the best air flow. Exceptionally, the biopsy specimens were taken from the narrowest cavity, when pathological changes were predominantly on this side.

One biopsy specimen was immersed in 3% glutaraldehyde in 0·1M cacodylate buffer, pH 7·4, at room temperature. After rinsing in 0·1M cacodylate buffer and 0·1M sucrose, the specimens were post-fixed in 1% osmium tetroxide in 0·1M cacodylate buffer, dehydrated in ethanol, embedded in Epon, cut in series of semi-thin sections (0·5–1 μm) and stained with toluidine blue. The second biopsy specimen was used for quantitative nickel analysis (Torjussen *et al.*, 1977).

*Blood and urine:* These were collected and analysed for nickel (Andersen, *et al.*, 1978).

### *Collection of relevant information*

Occupational history, including duration and type of nickel exposure, and smoking habits, were evaluated from a questionnaire and an interview. Persons who had stopped smoking less than one year before the start of the investigation, were recorded as smokers.

### *Procedures for histological examinations*

All sections were examined simultaneously by two of us (W.T. and L.A.S.) without access to any information on the specimen, and classified according to presence of:

*Normal respiratory epithelium.*—Pseudostratified or stratified. Ciliated columnar cells and goblet cells (Fig. 1).

*Stratified cuboidal epithelium.*—Cuboidal shape of cells from basal layer to surface. Loss of cilia (Fig. 2).

*Mixed stratified cuboidal/stratified squamous epithelium.*—Cuboidal cell layer covered by a thin layer of squamous cells. Cuboidal cell layer frequently loose and spongy (Fig. 3).

*Stratified squamous epithelium.*—Squamous cells demarcated by distinct cell borders. "Intercellular bridges" appear. Type I (Fig. 4) less thickened than Type II (Fig. 5), which has marked parakeratotic surface cells.

### *Characteristics noted*

*Hyperchromatic cell nuclei.*—Granular chromatin network with small chromocentres.

*Epithelial dysplasia.*—Arrangements of cells somewhat irregular. Tendency of loss of normal polarity towards surface. Cellular polymorphism with some variation in size of cells and nuclei. Hyperchromasia of cell nuclei (Fig. 6).

*Carcinoma* in situ.—Appreciable disarrangement of individual cells. Loss of polarity. Considerable cellular polymorphism. Hyperchromatic cell nuclei with dense chromatin and varying size (Fig. 7).

*Invasive carcinoma.*—Invasion of atypical cells into the stromal layer of the epithelium (Fig. 8).

The different characteristics of the surface epithelium were given scores from 0 to 7 according to Table II. Specimens with more than one type of epithelium were recorded according to the highest score.

TABLE II.—*Histological characteristics and scores for evaluation of nasal epithelium**

| Histological characteristics | Histological score |
|---|---|
| (a) Pseudostratified columnar epithelium | 0 |
| (b) Stratified cuboidal epithelium | 1 |
| (c) Mixed cuboidal/squamous epithelium | 2 |
| (d) Stratified squamous epithelium (Type I) | 3 |
| (e) Stratified squamous epithelium (Type II) | 4 |
| (f) Hyperchromatic nuclei (additional score) | 1 |
| (g) Epithelial dysplasia | 6 |
| (h) Carcinoma/carcinoma *in situ* | 7 |

* Maximum score from (a) to (f) is 5 points. Specimens with epithelial dysplasia or carcinoma were given 6 or 7 points, respectively, regardless of the scores from (a) to (f).

*Procedures for chemical analyses*

Measurements of nickel concentrations in nasal mucosa, plasma and urine were performed by atomic absorption techniques (Torjussen *et al.*, 1977; Andersen *et al.*, 1978). The results are reported elsewhere (Torjussen & Andersen, 1979) and used here only for statistical calculations (see below).

*Statistical methods*

In an attempt to explain the histological score (Y) from 0 to 7 by means of independent variables ($X_1, X_2 \ldots X_9$) the following model equation was applied on the material of active nickel workers and controls (n = 375):

$$Y = a + b_1X_1 + b_2X_2 + b_3X_3 + b_4X_4 + b_5X_5 + b_6X_6 + b_7X_7 + b_8X_8 + b_9X_9$$

Symbols:

a = Constant

$b_1 \ldots b_9$ = Regression coefficients

$X_1 = 1$ if the subject works with crushing/roasting/smelting; otherwise 0

$X_2 = 1$ if the subject works with electrolytic processes; otherwise 0

$X_3 = 1$ if the subject is involved in non-process work; otherwise 0.

$X_4$ = Individual age (years)

$X_5$ = Number of years from first employment at the plant

$X_6$ = Grams tobacco smoked per week

$X_7$ = $\mu$g Ni/100 g nasal mucosa, wet wt

$X_8$ = $\mu$g Ni/l plasma

$X_9$ = $\mu$g Ni/l urine

The explanatory values of each of the independent variables were tested by a forward stepwise multiple-regression analysis, calculating regression coefficients ($b_1 \ldots b_9$), and simple and partial correlation coefficients between histological score (Y) and each of the independent variables ($X_1 \ldots X_9$). The final equation was calculated from the model equation, and the significance of the correlation coefficients were tested by Student's *t* test.

Correlations between epithelial dysplasia (= 1; otherwise 0) and age, number of years from first nickel exposure, tobacco consumption and nickel concentrations in nasal mucosa, plasma and urine were also calculated. A probability level of less than 5% was required for significance.

### RESULTS

*Histopathology*

Fig. 1–8 present different types of epithelial changes in semi-thin sections stained with toluidine blue.

Among 390 biopsy samples, 368 (94·4%) showed more than one type of epithelium. Respiratory epithelium (Fig. 1) was found in 65% of controls, 57% of active nickel workers and in 33% of retired nickel workers (Table III). Stratified cuboidal epithelium (Fig. 2) was the most common type in both controls and active nickel workers, whereas stratified squamous epithelium (Fig. 4 & 5) was most frequently found in retired nickel workers.

Biopsy samples from 2 nickel workers, both employed 28 years, showed nasal carcinoma, one with squamous-cell carcinoma (Fig. 8), and one with an anaplastic

FIG. 1.—Stratified columnar epithelium with ciliated cells and goblet cells (×300: all figures have same magnification).

FIG. 2.—Stratified cuboidal epithelium with loss of ciliated cells.

FIG. 3.—Mixed stratified cuboidal/stratified squamous epithelium. The stratified cuboidal epithelium is covered by a thin layer of squamous cells.

FIG. 4.—Stratified squamous epithelium type I. Moderate thickened epithelium where the cells are demarcated by distinct light cell borders.

FIG. 5.—Stratified squamous epithelium type II. Thickened epithelium with budding of the epithelium into the stroma. Distinct surface layer of parakeratotic cells.

FIG. 6.—Epithelial dysplasia with disturbed polarity. The nuclei are hyperchromatic with marked variability in shape and size. The specimen is from a 62-year-old nickel worker, employed for 26 years at the Tankhouse department.

FIG. 7.—Carcinoma *in situ* with loss of polarity. Atypical squamous cells present in all layers of the epithelium. The variability in shape and size of the nuclei is pronounced. The section is from the same specimen as demonstrated in Fig. 8, but from an adjacent area.

FIG. 8.—Infiltrating squamous-cell carcinoma. The specimen is from a 61-year-old nickel worker employed at the Roasting/smelting department for 28 years.

TABLE III.—*Distribution of different types of nasal epithelium, dysplasia and carcinoma in nickel workers, retired nickel workers and in controls*

| Types of nasal eipthelium and epithelial changes | Number and percentage of subjects: Nickel workers (n=318) | Retired Nickel workers (n=15) | Controls (n=57) |
|---|---|---|---|
| Pseudostratified columnar | 180 (56·6%) | 5 (33·3%) | 37 (64·9%) |
| Stratified cuboidal | 297 (93·4%) | 11 (73·3%) | 52 (91·2%) |
| Mixed stratified cuboidal/stratified squamous | 204 (64·2%) | 10 (66·7%) | 12 (21·1%) |
| Stratified squamous | 146 (45·9%) | 14 (93·3%) | 16 (28·1%) |
| Hyperchromatic nuclei | 75 (23·6%) | 7 (46·7%) | 12 (21·1%) |
| Epithelial dysplasia | 38 (11·9%) | 7 (46·7%) | 1 (1·8%) |
| Carcinoma *in situ* | 1 (0·3%) | 0 | 0 |
| Carcinoma | 2 (0·6%) | 0 | 0 |

TABLE IV.—*Number of subjects in different histological groups, and average histological score according to working categories*

| Category of subject/work | No. of subjects | Histological score: 0–5 | 6 | 7 | Average score |
|---|---|---|---|---|---|
| Roasting/Smelting | 97 | 81 (84%) | 14 (12%) | 2 (2%) | 3·25 |
| Electrolysis | 144 | 128 (89%) | 16 (11%) | 0 | 3·01 |
| Non-process | 77 | 69 (90%) | 8 (10%) | 0 | 2·49 |
| Active nickel workers | 318 | 278 (87%) | 38 (12%) | 2 (0·6%) | 2·96 |
| Retired nickel workers | 15 | 8 (53%) | 7 (47%) | 0 | 4·93 |
| Controls | 57 | 56 (98%) | 1 (2%)* | 0 | 1·88 |

* Carpenter, 65 years old.

carcinoma. Carcinoma *in situ* was seen close to one of the invasive carcinomas (Fig. 7) but did not appear independently in any case. Epithelial dysplasia was found only in squamous epithelium of nickel-exposed individuals, with the exception of a 65-years-old carpenter from the control group (Table IV). The frequency of hyperchromatic nuclei was fairly equal in active nickel workers and in controls, but was higher in retired nickel workers (Table III).

The average histological score in controls is statistically significantly lower than in both active and retired nickel workers ($P < 0{\cdot}001$; Table IV). The difference in average score between active and retired nickel workers is also statistically significant ($P < 0{\cdot}001$). Disregarding the presence of epithelial dysplasia, and giving these specimens scores from 0 to 5 according to epithelial types alone, a significant difference between the groups is still present.

*Histopathology and age*

The correlation between epithelial dysplasia and age is statistically significant ($r = 0{\cdot}117$, $n = 375$; $P < 0{\cdot}05$). The youngest worker with epithelial dysplasia was 34 years old. Two men with carcinoma were both 61 years old.

Table V shows the average histological score related to various age intervals. The correlation between histological score and age is statistically significant ($P < 0{\cdot}003$; Table VII).

TABLE V.—*Average histological score according to age groups and working categories*

| Category of subject/work | Age groups (years): < 45 | 46–59 | > 60 | All |
|---|---|---|---|---|
| Roasting/smelting | 2·83 | 3·22 | 3·86 | 3·25 |
| Electrolysis | 2·64 | 3·15 | 3·24 | 3·01 |
| Non-process | 1·95 | 2·50 | 3·00 | 2·49 |
| Active nickel workers | 2·55 | 3·01 | 3·36 | 2·96 |
| Retired nickel workers | — | — | 4·93 | 4·93 |
| Controls | 1·78 | 2·13 | 1·60 | 1·88 |

TABLE VI.—*Average histological score according to smoking habits and working categories*

| Category of subject/work | Non-smokers | Smokers | All |
|---|---|---|---|
| Roasting/smelting | 2·65 | 3·53 | 3·25 |
| Electrolysis | 2·68 | 3·21 | 3·01 |
| Non-process | 2·14 | 2·83 | 2·49 |
| Active nickel workers | 2·50 | 3·24 | 2·96 |
| Retired nickel workers | 4·75 | 5·14 | 4·93 |
| Controls | 1·67 | 2·77 | 1·88 |

*Histopathology and duration of nickel exposure*

The correlation between epithelial dysplasia and number of years from first nickel exposure is not statistically significant ($r = 0{\cdot}078$, $n = 375$; $P > 0{\cdot}10$), whereas the correlation between histological score and the latter is ($P < 0{\cdot}001$; Table VII). Only 2/38 nickel workers with epithelial dysplasia had less than 10 years' employment at the plant; both working at the Roasting/Smelting Department.

TABLE VII.—*Simple and partial correlations between histological scores* ($Y$) *and some explaining variables* ($X_1$–$X_9$) *in* 375 *subjects*

| Correlation between Y and: | Correlation coefficients ($r$) Simple | Partial |
|---|---|---|
| Roasting/smelting ($X_1$) | 0·146** | 0·173** |
| Electrolysis ($X_2$) | 0·105* | 0·126* |
| Non-process work ($X_3$) | −0·094 | 0·079 |
| Age in years ($X_4$) | 0·155** | 0·163** |
| Years from first nickel exposure ($X_5$) | 0·179** | −0·012 |
| Grams tobacco per week ($X_6$) | 0·192** | 0·185** |
| $\mu$g Ni/100 g nasal mucosa (wet wt) ($X_7$) | −0·015 | −0·083 |
| $\mu$g Ni/l plasma ($X_8$) | 0·153** | 0·032 |
| $\mu$g Ni/l urine ($X_9$) | 0·141** | 0·025 |

* $P < 0{\cdot}05$. ** $P < 0{\cdot}01$.

*Histopathology and categories of work at the refinery*

Table IV shows the frequency of carcinoma, epithelial dysplasia and average histological score in nickel workers allocated to 3 different categories of work. The 2 men with carcinoma were employed at the Roasting/Smelting Department. No significant difference in frequency of epithelial dysplasia was found among the work categories. Seven out of 8 non-process workers with epithelial dysplasia had longer former employment as process workers, as compared with 27/69 without dysplasia. Ten electrolytic workers had more than one year's former employment at the Roasting/Smelting Department, and 2 of them had epithelial dysplasia.

The average histological scores are highest for roasting/smelting followed by those for electrolysis and non-process work. The difference in average score between roasting/smelting and electrolysis is not statistically significant ($P > 0{\cdot}10$), whereas the difference between each of these work categories and non-process work is ($P < 0{\cdot}001$ and $P < 0{\cdot}05$ respectively). The correlation between histological score and work category is statistically significant for roasting/smelting ($P < 0{\cdot}005$) and electrolysis ($P < 0{\cdot}04$), but not for non-process work ($P > 0{\cdot}10$; Table VII).

*Histopathology and tobacco smoking*

The percentage of smokers was nearly equal for active nickel workers and controls, but lower for retired nickel workers (Table VI). There is no statistically significant difference between the smoker's average tobacco consumption in active nickel workers and controls ($P > 0{\cdot}10$).

The 2 men with nasal carcinoma were both smokers. However, no correlation was found between tobacco smoking consumption and epithelial dysplasia ($r = 0{\cdot}046$, $n = 375$; $P > 0{\cdot}10$). The average histological score is, however, higher in smokers than in non-smokers, for both active and retired nickel workers and for controls (Table VI). Furthermore, the correlation between histological score and tobacco consumption is statistically significant ($P < 0{\cdot}0001$; Table VII).

*Histopathology and nickel in nasal mucosa, plasma and urine*

No significant correlation was found between epithelial dysplasia and nickel concentrations in nasal mucosa, plasma or urine ($P > 0{\cdot}10$; Table VII).

*Histological score and multiple regression analysis*

Table VII presents the simple and partial correlations between histological score and each of the independent variables. In the stepwise multiple-regression analysis the explanatory value of number of years from first nickel exposure ($X_5$) is reduced to the benefit of age ($X_4$). These two variables are, however, interrelated ($r = 0{\cdot}28$; $P < 0{\cdot}0001$), and number of years from first nickel exposure ($X_5$) was therefore preferred in the final equation.

Rejecting age ($X_4$) and the nickel concentrations ($X_7$, $X_8$ and $X_9$) from the model equation, the following final equation was calculated:

$$Y = 1{\cdot}59 + 0{\cdot}96X_1 + 0{\cdot}76X_2 + 0{\cdot}26X_3 + 0{\cdot}016X_5 + 0{\cdot}0063X_6$$

The multiple correlation coefficient between histological score and the variables included in the final equation is 0·32 ($P < 0{\cdot}0001$), which means that the equation can explain about 10% of the histological score.

DISCUSSION

In a preliminary study of histopathological changes of nasal mucosa in nickel workers (Torjussen *et al.*, 1979), carcinoma and epithelial dysplasia were found exclusively in process workers with at least 10 years' employment. Consequently, we have included all process workers with more than 8 years' employment in this study. For comparison, a randomly selected number of non-process workers, a group of retired nickel workers and a group of age-matched controls have been included.

The anterior curvature of the middle nasal turbinate forms a preferential area for dust particle deposition in the nasal mucosa (Hadfield, 1970) and is also frequently the origin for nickel-related nasal carcinomas (Virtue, 1972; Torjussen *et al.*, 1979). Biopsy samples taken from this site may therefore supply the most representative material for studies of local carcinogenic effects of chemical compounds on nasal mucosa.

From preliminary studies, applied plastic embedding was found superior to paraffin embedding for detailed studies of epithelial changes.

Oppikofer (1906) demonstrated the great variety of the surface epithelium on the anterior curvature of the middle nasal turbinate, which has recently been confirmed (Torjussen & Solberg, 1976; Torjussen *et al.*, 1979). The different types of non-dysplastic epithelium that are found in all groups of the present material may be regarded as non-specific reactions in the nasal mucosa. Our evaluation of the nasal mucosa was based on the assumption that long-lasting local influence of chemical and physical factors lead to gradual changes of the respiratory epithelium which, *via* stratified cuboidal and mixed stratified cuboidal/stratified squamous types, develops towards a fully stratified squamous epithelium. We further assumed that nickel-related invasive carcinoma of the nasal mucosa is preceded by epithelial dysplasia and carcinoma *in situ*, and that such changes might be detected in nickel-exposed individuals.

The finding of 2 nasal carcinomas among 318 active nickel workers confirms previous reports on the high incidence of the disease in nickel workers (Doll, 1958; Doll *et al.*, 1970, 1977; Mastromatteo, 1967; Pedersen *et al.*, 1973; Torjussen *et al.*, 1979). Nickel-related nasal carcinoma occurs several years after the start of employment (Morgan, 1958; Pedersen *et al.*, 1973; Torjussen *et al.*, 1979). Both cases in the present series were diagnosed after 28 years' employment.

As it seemed probable that nasal carcinoma is preceded by precancerous epithelial changes, we expected to find a high prevalence of such changes in our nickelexposed groups. However, except for carcinoma *in situ* in a specimen showing invasive carcinoma (Fig. 7), we did not observe obvious precancerous lesions. The lack of cases with carcinoma *in situ* may be accidental or due to the small size of our biopsy samples, leaving the possibility open that in some of the nickel-exposed workers carcinoma *in situ* might be present in other parts of the nasal mucosa. On the other hand, the widely accepted theory that carcinoma *in situ* usually precedes invasive carcinoma by several years (Thomas, 1973) may not be valid for the nasal mucosa, thus explaining a low prevalence of morphologically obvious precancerous lesions in this area.

As to the epithelial dysplasia among nickel workers in our study (Fig. 6), we do not consider the morphological changes so grave as frankly to indicate a premalignant state. Nevertheless, some of these changes may well represent early precancerous lesions. Our data clearly speak in favour of this assumption. Thus epithelial dysplasia was, with one exception, exclusively found in nickel workers. The only control subject with epithelial dysplasia belonged to another occupational group (woodworkers) with an increased incidence of nasal carcinoma (Hadfield, 1970; Andersen *et al.*, 1977). The precancerous character of nasal epithelial dysplasia cannot, however, be proved unless it is possible to demonstrate that such dysplasia develops into carcinoma *in situ* and finally into invasive carcinoma, like the states in the uterine cervix (Thomas, 1973). A close follow-up of the present material may in time provide such evidence.

Seven of the 8 registered non-process workers with epithelial dysplasia had their longest employment as process workers. Taking this into account and noting that all retired nickel workers with epithelial dysplasia had previously been process workers, epithelial dysplasia seems to be clearly connected to nickel process work. The fact that nickel process workers are exposed to the highest air-nickel concentrations at the plant implies that the amount of nickel exposure to the nasal mucosa is a probable causative factor for development of epithelial dysplasia.

Nickel concentrations in nasal mucosa are significantly higher in process workers than in non-process workers (Torjussen & Andersen, 1979) and to some degree also reflect the amounts of atmospheric nickel to which the workers are exposed. Thus a high concentration of mucosal nickel was particularly found in subjects from the Roasting/Smelting Department, although with great individual variations. These variations may explain the lack of significant correlation between mucosal nickel content and epithelial dysplasia in this study. Plasma and urine nickel reflects mainly the water-soluble part of the atmospheric nickel exposure (Torjussen & Andersen, 1979). This may be the reason for the lack of correlation between nickel concentrations in body fluids and epithelial dysplasia.

The results of present and previous studies (Torjussen *et al.*, 1979) show that epithelial dysplasia appears several years after the first nickel exposure. The incidence of dysplasia also increases with age and duration of nickel exposure, factors which are significantly interrelated. The notably high prevalence of epithelial dysplasia in retired nickel workers indicates persistence and possibly also increasing frequency of dysplastic changes in nasal mucosa after stopping active nickel refining work.

A causal relationship between tobacco smoking and nasal carcinoma has never been claimed. Both men with nasal carcinoma in our study had been smokers for years; however, no correlation between smoking habits and nasal epithelial dysplasia was found. Kreyberg (1978) indicated that tobacco smoking may have contributed to the increased incidence of lung cancer in nickel workers, whereas Pedersen *et al.* (1973; 1978), who collected their data from the same plant as the present work, were inconclusive on this point. Tobacco contains variable amounts of nickel, however, and cigarettes handrolled by nickel workers are considerably contaminated with nickel (Torjussen, 1979).

The data presented in this study show that the high prevalence of nasal epithelial dysplasia and carcinoma is clearly related to nickel-refining work. Animal experiments have proved that nickel subsulphide and nickel oxide are potent carcinogens (Gilman, 1962; Ottolenghi *et al.*, 1975; Yarita & Nettesheim, 1978), both being compounds that are common in the working atmosphere at the Roasting/Smelting Department. Both men with nasal carcinoma worked in this department. We believe that inhaled nickel compounds are the main carcinogens for nasal carcinoma in these workers, even though additional factors may contribute.

A complexity of factors seems to contribute to the transformation of respiratory nasal epithelium to squamous epithelium. The different factors analysed in our study are more or less interrelated, and a multiple regression analysis was made to find the factors that had independent explanatory values. Categories of work, number of years from first nickel exposure and tobacco consumption included in the final equation can, however, only explain about 10% of the histopathological variety. Other extrinsic factors, such as temperature, humidity, dusts or chemical compounds other than nickel in the environmental atmosphere, may be responsible for some of the remaining unexplained histopathological changes. Although the non-dysplastic epithelial changes are more pronounced in the nickel-exposed groups than in controls, they are obviously nonspecific and not only caused by nickel. Nevertheless, such nonspecific epithelial changes may still be essential steps in the development of epithelial dysplasia and nasal carcinoma. It should be noted that in this study epithelial dysplasia was exclusively found in squamous epithelium.

The health risk combined with nickel refining requires precautions to protect the employees. It is therefore necessary to work for the reduction of nickel exposure, since we do not know the safe threshold for nickel concentration in an occupational atmosphere. Subjects exposed to inevitably high nickel concentrations must be made to wear protective masks. Regular health controls should include roentgenograms of the chest to detect early pulmonary cancer. Nickel measurements in plasma and urine should be made at fixed intervals, along with examination of nasal biopsy specimens or cytological smears. Subjects with proven nasal epithelial dysplasia should be transferred to work with minimum nickel exposure and regularly followed up for early detection of malignant disease of the respiratory tract. The benefit of such a programme, carried out in the hope of reducing the occurrence of cancer, can only be evaluated in the future.

This investigation has been supported by grants from: Norsk Forening til Kreftens Bekjempelse, Oslo, Norway, Landsforeningen mot Kreft, Oslo, Norway, and Falconbridge Nikkelverk A/S, Kristiansand S, Norway.

### REFERENCES

ANDERSEN, H. C., ANDERSEN, I. & SOLGAARD, J. (1977) Nasal cancers, symptoms and upper airway function in woodworkers. *Br. J. Ind. Med.*, **34**, 201.

ANDERSEN, I., TORJUSSEN, W. & ZACHARIASEN, H. (1978) Analysis for nickel in plasma and urine by electrothermal atomic absorption spectrometry, with sample preparation by protein precipitation. *Clin. Chem.*, **24**, 1198.

DOLL, R. (1958) Cancer of the lung and nose in nickel workers. *Br. J. Ind. Med.*, **15**, 217.

DOLL, R., MATHEWS, J. D. & MORGAN, L. G. (1977) Cancers of the lung and nasal sinuses in nickel workers: a reassessment of the period of risk. *Br. J. Ind. Med.*, **34**, 102.

DOLL, R., MORGAN, L. G. & SPEIZER, F. E. (1970) Cancer of the lung and nasal sinuses in nickel workers. *Br. J. Cancer*, **24**, 623.

GILMAN, J. P. W. (1962) Metal carcinogenesis. II. A study on the carcinogenic activity of cobalt, iron, and nickel compounds. *Cancer Res.*, **22**, 158.

HADFIELD, E. H. (1970) A study of adenocarcinoma of the paranasal sinuses in woodworkers in the furniture industry. *Ann. R. Coll. Surg. Engl.*, **46**, 301.

IARCH (1976) Monographs on the evaluation of the carcinogenic risk of chemicals to man. Nickel and nickel compounds. In *Cadmium and Nickel.* Lyon, France: International Agency for Research on Cancer. p. 75.

KREYBERG, L. (1978) Lung cancer in workers in a nickel refinery. *Br. J. Ind. Med.*, **35**, 109.

MASTROMATTEO, E. (1967) Nickel: A review of its occupational health aspects. *J. Occup. Med.*, **9**, 127.

MORGAN, J. G. (1958) Some observations on the incidence of respiratory cancer in nickel workers. *Br. J. Ind. Med.*, **15**, 224.

NIOSH (1977) *Criteria for a recommended standard. Occupational exposure to inorganic nickel.* Washington: Department of Health, Education, and Welfare.

OPPIKOFER, E. (1906) Beitrage zur normalen und pathologischen Anatomie der Nase und ihren Nebenhölen. *Archiv. Laryngol. Rhinol.*, **19**, 28.

OTTOLENGHI, A. D., HASEMAN, J. K., PAYNE, W. W., FALK, H. L. & MACFARLAND, H. N. (1975) Inhalation studies of nickel sulfide in pulmonary carcinogenesis of rats. *J. Natl Cancer Inst.*, **54**, 1165.

PEDERSEN, E., ANDERSEN, A. & HØGETVEIT, A. C. (1978) Second study of the incidence and mortality of cancer of respiratory organs among workers at a nickel refinery. *Ann. Clin. Lab. Sci.*, **8**, 503. (Abst.).

PEDERSEN, E. A., HØGETVEIT, A. C. & ANDERSEN, A. (1973) Cancer of respiratory organs among workers at a nickel refinery in Norway. *Int. J. Cancer*, **12**, 32.

SUNDERMAN, F. W., JR (1977) A review of the metabolism and toxicology of nickel. *Ann. Clin. Lab. Sci.*, **7**, 377.

SUNDERMAN, F. W., JR, COULSTON, F., EICHHORN, G. I. & 4 others (1975) *Nickel.* Washington: Academy of Sciences.

THOMAS, D. B. (1973) An epidemiologic study of carcinoma *in situ* and squamous dysplasia of the uterine cervix. *Am. J. Epidemiol.*, **98**, 10.

TORJUSSEN, W. (1979) Rhinoscopical findings in nickel workers, with special emphasis on the influence of nickel exposure and smoking habits. *Acta Otolaryngol.* (in press).

TORJUSSEN, W., ANDERSEN, I. & ZACHARIASEN, H. (1977) Nickel content in human palatine tonsils: Analysis of small tissue samples by flameless atomic absorption spectrophotometry. *Clin. Chem.*, **23**, 1018.

TORJUSSEN, W. & SOLBERG, L. A. (1976) Histological findings in the nasal mucosa of nickel workers. A preliminary report. *Acta Otolaryngol.*, **82**, 266.

TORJUSSEN, W., SOLBERG, L. A. & HØGETVEIT, A. C. (1979) Histopathological changes of nasal mucosa in nickel workers: A pilot study. *Cancer* (in press).

TORJUSSEN, W. & ANDERSEN, I. (1979) Nickel concentrations in nasal mucosa, plasma and urine in active and retired nickel workers. *Ann. Clin. Lab. Sci.* (in press).

VIRTUE, J. A. (1972) The relationship between refining of nickel and cancer of the nasal cavity. *Can. J. Otolaryngol.*, **1**, 37.

YARITA, T. & NETTESHEIM, P. (1978) Carcinogenicity of nickel subsulfide for respiratory tract mucosa. *Cancer Res.*, **38**, 3140.